# Association of macular pigment optical density with retinal layer thicknesses in eyes with and without manifest primary open-angle glaucoma

**DOI:** 10.1136/bmjophth-2023-001331

**Published:** 2023-10-27

**Authors:** Thomas Lawler, J A Mares, Zhe Liu, Catherine Thuruthumaly, Tyler Etheridge, Thasarat S Vajaranant, Amitha Domalpally, Billy R Hammond, Robert B Wallace, Lesley F Tinker, Marine Nalbandyan, Barbara E K Klein, Yao Liu

**Affiliations:** 1 Department of Nutritional Sciences, College of Agricultural & Life Sciences, University of Wisconsin, Madison, Wisconsin, USA; 2 Department of Ophthalmology and Visual Sciences, University of Wisconsin, Madison, Wisconsin, USA; 3 Ophthalmology & Visual Sciences, Medical College of Wisconsin, Milwaukee, Wisconsin, USA; 4 Department of Ophthalmology & Visual Sciences, University of Illinois, Chicago, Illinois, USA; 5 Department of Psychology, University of Georgia, Athens, Georgia, USA; 6 Department of Epidemiology, University of Iowa, Iowa City, Iowa, USA; 7 Department of Cancer Prevention, Fred Hutchinson Cancer Center, Seattle, Washington, USA

**Keywords:** glaucoma, epidemiology, optic nerve, retina, imaging

## Abstract

**Objective:**

To investigate associations between baseline macular pigment optical density (MPOD) and retinal layer thicknesses in eyes with and without manifest primary open-angle glaucoma (POAG) in the Carotenoids in Age-Related Eye Disease Study 2 (CAREDS2).

**Methods and analysis:**

MPOD was measured at CAREDS baseline (2001–2004) via heterochromatic flicker photometry (0.5° from foveal centre). Peripapillary retinal nerve fibre layer (RNFL), macular ganglion cell complex (GCC), ganglion cell layer (GCL), inner plexiform layer (IPL), and RNFL thicknesses were measured at CAREDS2 (2016–2019) via spectral-domain optical coherence tomography. Associations between MPOD and retinal thickness were assessed using multivariable linear regression.

**Results:**

Among 742 eyes (379 participants), manifest POAG was identified in 50 eyes (32 participants). In eyes without manifest POAG, MPOD was positively associated with macular GCC, GCL and IPL thicknesses in the central subfield (P-trend ≤0.01), but not the inner or outer subfields. Among eyes with manifest POAG, MPOD was positively associated with macular GCC, GCL, IPL and RNFL in the central subfield (P-trend ≤0.03), but not the inner or outer subfields, and was positively associated with peripapillary RNFL thickness in the superior and temporal quadrants (P-trend≤0.006).

**Conclusion:**

We observed a positive association between MPOD and central subfield GCC thickness 15 years later. MPOD was positively associated with peripapillary RNFL superior and temporal quadrant thicknesses among eyes with manifest POAG. Our results linking low MPOD to retinal layers that are structural indicators of early glaucoma provide further evidence that carotenoids may be protective against manifest POAG.

**WHAT IS ALREADY KNOWN ON THIS TOPIC**

- Small, cross-sectional case–control studies have shown an association between macular pigment and ganglion cell complex thickness.

**WHAT THIS STUDY ADDS**

- We observed a positive association between baseline macular pigment optical density and ganglion cell complex thickness within the central subfield measured 15 years later among healthy and glaucomatous eyes in a large cohort of older women.

**HOW THIS STUDY MIGHT AFFECT RESEARCH, PRACTICE OR POLICY**

- This study supports continued development of clinical trials to determine whether interventions to increase macular pigment may prevent glaucoma development or progression.

## Introduction

Primary open-angle glaucoma (POAG) is a leading cause of irreversible blindness,^1^ estimated to affect 44 million adults globally.^2^ POAG is characterised by death of retinal ganglion cells (RGCs) and their axons in the retinal nerve fibre layer (RNFL).^3^ The highest density of RGCs exists in the macula, which is increasingly recognised as a site of early glaucoma pathogenesis.^4^ Thinning of the macular ganglion cell complex (GCC) and peripapillary RNFL can occur early in glaucoma, prior to detectable visual field defects.^4^ Consequently, interventions to prevent macular GCC thinning may prevent glaucomatous vision loss.

There have been conflicting reports regarding whether POAG is associated with lower levels of dietary carotenoids lutein and zeaxanthin (L/Z), which comprise macular pigment.^5–7^ L/Z are antioxidants that accumulate in neural tissues throughout the brain and retina,^8 9^ with highest density in the fovea.^10^ Macular pigment may protect RGCs and their axons by neutralising reactive-oxygen species and providing structural support in cell membranes.^11 12^ These postulated neuroprotective effects are consistent with growing evidence that macular pigment and greater dietary L/Z are positively associated with cognition^13^ and lower risk of age-related macular degeneration^11 14 15^ and Alzheimer’s disease.^16^ Thus, macular pigment may be a novel POAG risk factor for intervention, as it can be measured non-invasively and modified via diet or nutritional supplementation.^11^

Several studies have reported a positive relationship between macular pigment optical density (MPOD) and thickness of retinal layers affected in early-stage glaucoma.^5 6 17^ However, these studies have been predominantly small and cross-sectional. In this study, we investigated the association between baseline MPOD and thickness of the macular GCC and peripapillary RNFL approximately 15 years later among participants in the Carotenoids in Age-Related Eye Disease Study 2 (CAREDS2), an ancillary study of the prospective Women’s Health Initiative (WHI) Observational Study. Since glaucomatous neurodegeneration significantly reduces macular GCC and peripapillary RNFL thickness,^1 4^ we examined associations with MPOD separately in eyes with and without manifest POAG. We tested the hypothesis that lower MPOD would be associated with thinner macular GCC and peripapillary RNFL thicknesses 15 years later.

## Materials and methods

### CAREDS study design and sample

CAREDS is an ancillary study of the WHI, a multicentre prospective study of postmenopausal women in the USA.^18^ The CAREDS baseline study design and recruitment process has been previously described.^19^ CAREDS was originally designed to study the relationship between diet and lifestyle with macular degeneration and cataract development. Glaucoma measures were subsequently added in CAREDS2 and were not assessed at CAREDS baseline.

At CAREDS baseline (2001–2004), 2005 women were recruited from three WHI study sites (Iowa City, Iowa; Madison, Wisconsin; Portland, Oregon) and completed either: an in-person study visit with questionnaires on demographics, medical history, dietary intake and supplement use (n=1894) or questionnaires only (n=111). In the follow-up CAREDS2 study (2016–2019) (n=685 participants), 487 completed the CAREDS2 in-person study visit and questionnaires, while 198 completed questionnaires only. Those who did not participate in CAREDS2 (n=1320) were either deceased (48.4%), had been lost to follow-up or refused further contact (35.9%) or either declined participation or could not be contacted (15.7%). All participants provided written informed consent. Patients were involved in the design of our research in that we obtained input and a letter of support from the University of Wisconsin Glaucoma Patient Support Group. This study was approved by the University of Wisconsin-Madison Health Sciences Institutional Review Board (IRB) and was conducted in accordance with the tenets of the Declaration of Helsinki.

In this study, we analysed data from participants who attended the CAREDS2 in-person study visit. Exclusion criteria included missing MPOD in both eyes at CAREDS baseline or missing retinal thickness measures in both eyes, axial length >26 mm, presence of advanced age-related macular degeneration (ie, geographic atrophy or neovascular disease), insufficient data to adjudicate manifest POAG or either narrow angles or secondary glaucoma at CAREDS2 (online supplemental figure 1).

10.1136/bmjophth-2023-001331.supp1 Supplementary data

### Assessment of MPOD

MPOD at CAREDS baseline (2001–2004) was measured in both eyes via customised heterochromatic flicker photometry (Macular Metrics I, LLC, Rehoboth, Massachusetts, USA), a valid and reproducible psychophysical technique for MPOD measurement in older adults.^20^ MPOD was measured using a table-top densitometer similar to the device initially described by Wooten *et al*.^21^ In brief, MPOD was measured in the fovea at four targets, including 0.25°, 0.50°, 1.00° and 1.75° from the foveal centre, relative to a 7° reference measure. Measurements were completed first in the right eye, and then in the left eye (0.25° and 0.50° targets only). For each target, the MPOD measurement was calculated from five separate determinations, using a blue light-emitting diode with a peak wavelength of 460 nm, corresponding to the maximum absorption spectrum of macular pigment. For each participant, the flicker rate for MPOD testing was adjusted based on the individual’s critical flicker frequency at the foveal and parafoveal targets, which were measured prior to MPOD testing. A detailed protocol for MPOD measurement in CAREDS has been published previously.^22^ MPOD at 0.5° (central fovea) was utilised as the primary exposure for the analysis, as this target has the highest ratio of between-individual to within-individual variability.^22^

### Assessment of retinal layer thicknesses

Macular volume and peripapillary RNFL thickness scans were obtained at the CAREDS2 in-person study visit via spectral-domain optical coherence tomography (SD-OCT) imaging using the Heidelberg Spectralis (Heidelberg Spectralis, Heidelberg Engineering, Heidelberg, Germany). All SD-OCT scans were obtained by a certified photographer following a Wisconsin Reading Centre (WRC)-approved protocol. SD-OCT scans, including volume scans centred on the macula and peripapillary RNFL thickness scans were obtained from 473 CAREDS2 participants (n=946 eyes). Segmentation of retinal layers in the macula was generated using Heidelberg Spectralis software (V.1.9.13.0). Manual adjustment for segmentation error was completed by masked WRC-certified graders. A quality score of 20 or higher was required to be considered acceptable for inclusion. Images from 40 eyes were excluded due to the presence of poor signal strength, imaging artefacts or other ocular pathology that made segmentation unreliable.^23^ Additional details pertaining to the macular SD-OCT imaging and segmentation in CAREDS2 have been published previously.^23^ For the peripapillary RNFL, one circular RNFL scan was obtained consisting of 1536 A-Scans at 12° at high resolution with a frame rate of 100.

**Figure 1:**
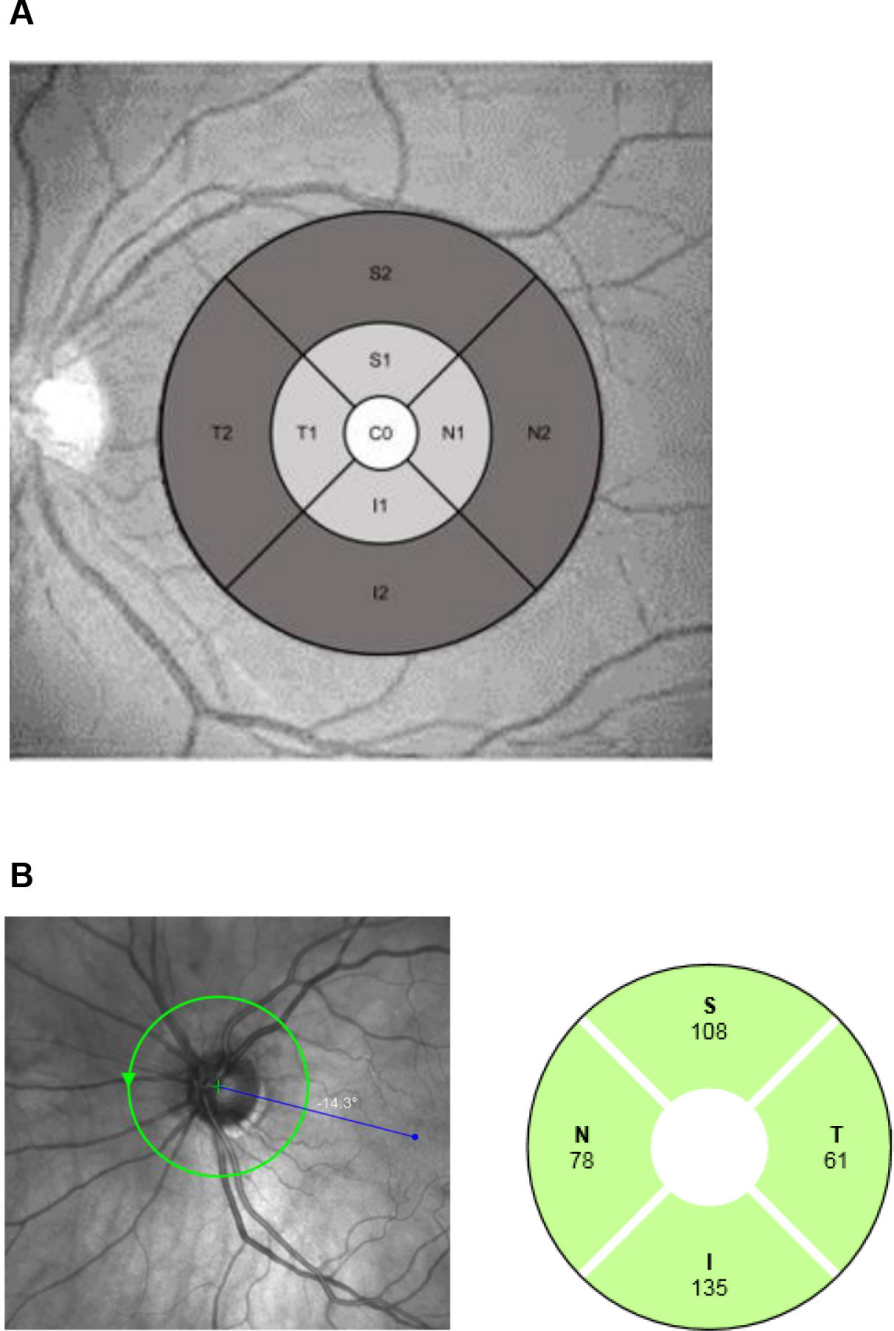
Maps of macular subfields* (A) and peripapillary retinal nerve fibre layer (B) thickness measurements using spectral-domain optical coherence tomography. *White: central subfield, light grey: inner subfield, dark grey: outer subfield. C0, central subfield; I, inferior quadrant; I1, inferior quadrant, inner subfield; I2, inferior quadrant, outer subfield; N, nasal quadrant; N1, nasal quadrant, inner subfield; N2, nasal quadrant, outer subfield; RNFL, retinal nerve fibre layer; S, superior quadrant; S1, superior quadrant, inner subfield; S2, superior quadrant, outer subfield; T, temporal quadrant; T1, temporal quadrant, inner subfield; T2, temporal quadrant, outer subfield.

Macular thickness measurements were obtained for the RNFL, ganglion cell layer (GCL) and inner plexiform layer (IPL) for each subfield of the ETDRS (Early Treatment of Diabetic Retinopathy Study) grid, including the central (1 mm diameter), inner (3 mm diameter) and outer subfields (6 mm diameter) (figure 1A). The inner and outer macular subfield thicknesses were calculated as the average of the inferior, superior, nasal and temporal quadrants within each subfield, respectively. GCC was calculated as the sum of the measurements for macular RNFL, GCL and IPL thickness. Peripapillary RNFL thickness measurements were obtained for the inferior, superior, nasal and temporal quadrants, as well as the average of all quadrants (figure 1B).

### Assessment of ocular characteristics

Ocular characteristics were evaluated by trained examiners at the CAREDS2 in-person study visit. These included intraocular pressure (IOP) (Tono-Pen, Reichert Inc, Depew, New York, USA), axial length (average of three measurements) (Gilras GRU-5000 A Biometer, US Ophthalmic, Coral, Florida, USA) and corneal pachymetry (PachPen, Accutome, Inc, Malvern, Pennsylvania, USA). A slit-lamp examination was conducted to ascertain whether an intraocular lens was implanted. Stereoscopic 30° digital colour fundus photographs of the optic nerve (Topcon TRC-DX50, Tokyo, Japan) were obtained by a WRC-certified photographer following pupil dilation with 2.5% phenylephrine and 1% tropicamide. The vertical cup-to-disc ratio was measured by a WRC-certified grader via IMAGEnet software (*IMAGEnet 6, Topcon Healthcare, Oakland, NJ, USA*) and the presence of disc haemorrhage or notching were identified following a standard protocol.^24^

### Adjudication of manifest POAG

Detailed medical records were requested from eye care providers for participants who had at least one glaucoma risk factor. These risk factors including self-reported glaucoma or self-reported use of glaucoma medications, vertical cup-to-disc ratio ≥0.6 in either eye, vertical cup-to-disc asymmetry ≥0.2, disc haemorrhage or notching, IOP ≥22 mm Hg or peripapillary RNFL thickness <5th percentile for the average of all quadrants or for the inferior or superior quadrants. Medical records included visual field tests, peripapillary RNFL OCT imaging, fundus photographs and clinic notes. If a patient had either: no history of reproducible glaucomatous visual field defects or had unreliable visual fields (eg, >33% fixation losses, >25% false positives or >25% false negatives) or the most recent reliable visual fields were completed more than 1 year ago, the participant was invited to return for visual field testing. Humphrey visual field testing (Carl Zeiss Meditec, Inc, Jena, Germany) was completed in each eye using the SITA (Swedish Interactive Threshold Algorithm)-Standard 24-2 testing algorithm. Visual field testing was completed sequentially in the right eye and then left eye by a certified, trained examiner using each participants’ near refractive correction for each eye. Visual field testing was repeated for unreliable visual fields or if a rim artefact was suspected by the examiner.

Two fellowship-trained glaucoma specialists (YL and CT) who were masked to baseline MPOD measurements completed adjudication of manifest POAG status based on medical records review, visual field testing, fundus photography, SD-OCT imaging and ocular examination characteristics from CAREDS2. Manifest POAG was defined based on similar criteria as used in the Nurses’ Health Study, including the presence of glaucomatous visual field defects (ie, temporal, nasal, arcuate or paracentral).^25^ Defects were reproducible from at least one prior visual field test, were not related to other ocular conditions and were consistent with the pattern of optic nerve thinning from stereoscopic optic disc photographs and/or peripapillary RNFL OCT measurements. Disagreements on manifest POAG diagnosis were resolved by achieving consensus on joint review of participant data.

### Statistical analysis

Age-adjusted multivariable regression models were used to assess associations between covariates and MPOD at CAREDS baseline, as well as manifest POAG status at CAREDS2. To assess potential survival bias, we also compared baseline characteristics among CAREDS participants (n=2005) who were included and those who were excluded from the analysis.

We used multiple linear regression to investigate associations between MPOD at CAREDS baseline and retinal layer thickness measurements approximately 15 years later at CAREDS2 among eyes with and without manifest POAG. Least-square means were calculated by quartile of MPOD, and β-coefficients and P-trends were calculated per one SD increase in MPOD as a continuous variable. All models were first adjusted for age and then other potential confounding covariates based on previously reported associations with MPOD in CAREDS and/or plausible causal associations with glaucoma or retinal thickness.^19^ Covariates were added individually to the age-adjusted model and retained if there was evidence of confounding (ie, change in the β-coefficients ≥10%). Only axial length met this criterion, and thus the final model included adjustment for age and axial length. Generalised estimating equations were used to account for correlations between eyes. A sensitivity analysis was conducted to exclude participants (approximately 16%) who reported using L/Z supplements (≥1 mg/day) prior to CAREDS2.

All participants had MPOD measurement in the right eye. For participants with missing baseline MPOD values in the left eye (n=17, 4.5%), the missing value was replaced with corresponding right eye value, as MPOD has high intereye correlation.^22^ Missing values for covariates were rare (approximately 1%–2% of observations) and were replaced using the median value. All analyses were conducted using SAS V.9.4 (Cary, North Carolina, USA). Statistical significance was set at P-trend <0.05.

## Results

We included 742 eyes from 379 women in the analysis (online supplemental figure 1). The median age at CAREDS baseline was 65 years (range: 55–81 years) (table 1). Participants were predominantly white (97.9%) and non-Hispanic (99.5%) and had completed some college or vocational education (87.1%). MPOD at CAREDS baseline (0.5°, right eye) was associated with intraocular lens implantation (P-trend=0.01), larger waist circumference (P-trend <0.001) and higher body mass index (BMI) (P-trend <0.001). Those excluded from the analysis were slightly older, had lower levels of educational attainment and income, were slightly more likely to have an intraocular lens, to smoke, have diabetes, have a larger waist circumference and have a higher BMI (online supplemental table 1).

10.1136/bmjophth-2023-001331.supp3 Supplementary data

**Table 1:** Participant characteristics by quartile of MPOD at CAREDS baseline (n=379)

Variable (mean±SD or percentage)	Full sample (n=379)	MPOD (optical density units), 0.5°				P-trend
		Quartile 1 (0.00–0.24)	Quartile 2 (0.24–0.38)	Quartile 3 (0.39–0.52)	Quartile 4 (0.52–1.00)	
Age (years)	65.4±0.3	64.4±0.6	65.9±0.6	65±0.6	66.3±0.6	0.10
Race						
Asian	0.8%	1.2%	0.0%	1.5%	1.0%	0.21
Black	0.8%	2.3%	1.2%	0.0%	0.0%	
White	97.9%	95.7%	98.8%	97.1%	99.0%	
More than one race	0.3%	0.0%	0.0%	1.5%	0.0%	
Unknown/not reported	0.3%	0.8%	0.0%	0.0%	0.0%	
Ethnicity						
Non-Hispanic	99.5%	100.0%	100.0%	99.1%	99.2%	0.55
Hispanic	0.5%	0.0%	0.0%	0.9%	0.8%	
Education						
High school graduate or less	12.9%	16.8%	10.4%	13.0%	12.9%	0.14
College or vocational training	48.0%	47.1%	56.3%	46.5%	37.5%	
Post college	39.1%	36.0%	33.3%	40.6%	49.6%	
Household Income						
<US$75 000	72.0%	73.6%	68.0%	75.2%	70.8%	0.51
>US$75 000	28.0%	26.4%	32.0%	24.8%	29.2%	
Pack years smoked						
Non-smoker	58.0%	52.6%	60.9%	58.7%	61.6%	0.18
<7 pack years	24.5%	28.1%	23.0%	21.3%	26.7%	
≥7 pack years	17.4%	19.3%	16.1%	20.0%	11.7%	
Intraocular lens implantation*	6.6%	11.4%	6.1%	7.8%	2.7%	0.01
Intraocular pressure (mm Hg)*	14.4±0.2	14.1±0.3	14.4±0.3	14.4±0.3	14.8±0.3	0.11
Axial length (mm)*	23.6±0.1	23.6±0.1	23.6±0.1	23.8±0.1	23.5±0.1	0.83
Corneal thickness (µm)*	558.2±1.9	556.2±3.8	560.7±3.7	556.1±3.7	559.7±3.7	0.61
Waist circumference (in.)	34.1±0.3	35.5±0.5	34.3±0.5	33.8±0.5	32.7±0.5	<0.001
Body mass index (kg/m^2^)	27.5±0.3	29.2±0.5	27.6±0.5	26.8±0.5	26.2±0.5	<0.001
Self-reported hypertension	21.4%	28.2%	18.1%	22.3%	17.2%	0.09
Self-reported diabetes	3.7%	3.4%	5.0%	2.6%	2.9%	0.46

*Values for ocular characteristics are shown only for the right eye.

CAREDS, Carotenoids in Age-Related Eye Disease Study; MPOD, macular pigment optical density.

Manifest POAG was identified in 50 eyes (6.7%) from 32 participants. Participants with manifest POAG were slightly older, more likely to be a non-smoker, to have an intraocular lens and to have lower MPOD at CAREDS baseline (online supplemental table 2). Among participants with manifest glaucoma (n=32), the average visual field mean deviation in the worse eye was −5.85 dB (66% mild, MD ≥−6 dB, 28% moderate, MD <−6 dB and ≥−12 dB, and 6% severe, <−12 dB, following the Hodapp-Parrish-Anderson glaucoma severity classification).^26^ Compared with eyes without manifest POAG, those with manifest POAG had thinner peripapillary RNFL thicknesses in all quadrants (p<0.001) (table 2). Eyes with manifest POAG had significantly thinner macular RNFL, GCL, IPL and total GCC thickness (p≤0.006) in the inner and outer subfields. Modestly lower macular RNFL (p=0.07) and total GCC thickness (p=0.09) in the central subfield were also observed for glaucomatous eyes.

**Table 2:** Retinal layer thickness* by manifest POAG status at CAREDS2

Retinal layer thickness* (μm, mean±SE)	No manifest POAG (n=692 eyes)	Manifest POAG (n=50 eyes)	β (SE)	P value
Peripapillary RNFL				
Average	93.4±0.5	72.5±1.7	20.8±1.8	<0.001
Inferior	120.8±0.9	83.7±3.1	37.1±3.3	<0.001
Superior	110.7±0.8	85.3±2.5	25.5±2.6	<0.001
Nasal	71.6±0.6	58.6±2.3	13.0±2.3	<0.001
Temporal	70.5±0.7	62.5±1.9	8.0±2.0	<0.001
Macular RNFL				
Central	13.2±0.1	12.1±0.6	1.1±0.6	0.07
Inner	23.6±0.1	22.4±0.4	1.2±0.4	0.006
Outer	39.6±0.3	32.3±1.0	7.4±1.1	<0.001
Macular GCL				
Central	16.1±0.2	15.1±1.0	1.1±1.0	0.28
Inner	46.2±0.3	38.7±1.3	7.5±1.4	<0.001
Outer	30.3±0.2	26.0±0.6	4.3±0.7	<0.001
Macular IPL				
Central	21.4±0.2	20.7±1.1	0.7±1.1	0.51
Inner	38.1±0.2	34.0±0.9	4.1±0.9	<0.001
Outer	25.5±0.1	23.1±0.3	2.5±0.4	<0.001
Macular GCC				
Central	50.5±0.5	46.6±2.2	3.8±2.3	0.09
Inner	107.8±0.5	95.0±2.5	12.7±2.5	<0.001
Outer	95.3±0.5	81.5±1.8	13.8±1.9	<0.001

*Adjusted for age and axial length.

CAREDS, Carotenoids in Age-Related Eye Disease Study; GCC, ganglion cell complex; GCL, ganglion cell layer; IPL, inner plexiform layer; POAG, primary open angle glaucoma; RNFL, retinal nerve fiber layer.

In eyes without manifest POAG, MPOD at CAREDS baseline was not associated with peripapillary RNFL thickness (P-trend ≥0.26) nor with macular RNFL thickness (P-trend ≥0.13) (table 3). MPOD was positively associated with macular IPL (P-trend <0.001), GCL (P-trend=0.01) and total GCC (P-trend=0.003) thickness in the central subfield, but not the inner or outer subfields (P-trend ≥0.15) (online supplemental figure 2). In the sensitivity analysis, excluding eyes from L/Z supplement users, there were no significant changes in these results (online supplemental table 3).

10.1136/bmjophth-2023-001331.supp2 Supplementary data

**Table 3:** Retinal layer thickness* by quartile of MPOD at CAREDS baseline among eyes without manifest POAG (n=347 participants)

Retinal layer thickness* (μm, mean±SE)	Number of eyes	MPOD-CAREDS baseline, 0.5° (optical density units)				β±SE (1−SD increase)	P*-*trend
		Quartile 1 (0.00–0.23)	Quartile 2 (0.23–0.38)	Quartile 3 (0.38–0.51)	Quartile 4 (0.51–1.00)		
Peripapillary RNFL							
Average	631	93.9±1.0	93.6±1.0	92.8±1.0	93.2±0.9	0.3±0.5	0.53
Inferior	631	120.9±1.4	121.2±1.5	120.1±1.6	121.1±1.7	0.2±0.7	0.77
Superior	631	111.6±1.5	110.9±1.4	109.8±1.4	110.9±1.4	0.2±0.8	0.80
Nasal	631	72.3±1.2	72.3±1.2	70.7±1.2	71.2±1.1	0.7±0.6	0.26
Temporal	631	71.4±1.3	70.3±1.3	70.8±1.2	69.7±1.2	0.4±0.6	0.50
Macular RNFL							
Central	668	13.2±0.3	12.9±0.2	13.1±0.2	13.7±0.2	0.2±0.1	0.13
Inner	668	23.9±0.3	23.5±0.3	23.5±0.2	23.6±0.2	0.1±0.1	0.53
Outer	666	40.1±0.6	39.1±0.5	39.7±0.5	39.6±0.5	0.0±0.3	0.88
Macular GCL							
Central	667	15.7±0.5	15.6±0.5	16.2±0.3	17.2±0.4	0.5±0.2	0.01
Inner	668	46.0±0.5	46.0±0.5	46.3±0.5	46.6±0.4	0.3±0.2	0.26
Outer	665	30.2±0.3	30.1±0.3	30.5±0.3	30.3±0.3	0.1±0.1	0.38
Macular IPL							
Central	669	20.9±0.4	20.8±0.3	21.4±0.3	22.4±0.3	0.6±0.2	<0.001
Inner	669	38.0±0.3	38.0±0.3	38.1±0.3	38.4±0.2	0.2±0.1	0.23
Outer	668	25.4±0.2	25.5±0.2	25.6±0.2	25.7±0.2	0.1±0.1	0.15
Macular GCC							
Central	656	49.2±1.0	48.9±1.0	50.6±0.8	53.1±0.8	1.3±0.4	0.003
Inner	657	107.6±0.9	107.4±1.0	107.8±1.0	108.5±0.7	0.4±0.4	0.35
Outer	655	95.5±0.8	94.7±0.9	95.6±1.0	95.6±0.8	0.3±0.4	0.50

*Adjusted for age and axial length.

CAREDS, Carotenoids in Age-Related Eye Disease Study; GCC, ganglion cell complex; GCL, ganglion cell layer; IPL, inner plexiform layer; MPOD, macular pigment optical density; POAG, primary open-angle glaucoma; RNFL, retinal nerve fibre layer.

Among eyes with manifest POAG, MPOD was positively associated with macular RNFL, IPL, GCL and GCC thickness in the central subfield (P-trend ≤0.03), but not the inner or outer subfields (P-trend ≥0.15) (table 4) (online supplemental figure 2). MPOD was also positively associated with peripapillary RNFL thickness in the superior (P-trend=0.005) and temporal quadrants (P-trend=0.006), but not in the average, inferior or nasal quadrants (P-trend ≥0.11). After excluding eyes from L/Z supplement users, the association with GCL thickness in the central subfield remained statistically significant, while associations with the macular IPL thickness (P-trend=0.09) and total GCC thickness (P-trend=0.07) were somewhat attenuated (online supplemental table 4).

**Table 4:** Retinal layer thickness* by quartile of MPOD at CAREDS baseline among eyes with manifest POAG (n=32 participants)

Retinal layer thickness* (μm, mean±SE)	Number of eyes	MPOD-CAREDS baseline, 0.5° (optical density units)				β±SE (1−SD increase)	P-trend
		Quartile 1 (0.00–0.07)	Quartile 2 (0.08–0.30)	Quartile 3 (0.32–0.48)	Quartile 4 (0.48–0.81)		
Peripapillary RNFL							
Average	40	67.0±3.9	73.2±2.1	75.3±2.4	72.2±3.2	2.3±1.4	0.11
Inferior	40	75.5±6.0	89.8±4.9	89.6±5.7	72.6±5.9	0.0±2.8	0.99
Superior	40	75.7±5.2	80.5±3.2	94.1±3.6	90.0±3.6	6.0±2.1	0.005
Nasal	40	61.0±4.0	57.6±3.2	56.6±5.0	58.0±3.5	0.8±2.0	0.69
Temporal	40	56.1±3.4	64.9±2.4	60.7±2.4	68.7±3.5	4.0±1.5	0.006
Macular RNFL							
Central	45	11.0±0.7	12.6±1.0	10.6±1.1	14.0±1.1	1.1±0.5	0.03
Inner	45	21.2±0.7	24.1±0.8	22.5±0.5	22.4±0.6	0.3±0.3	0.30
Outer	45	29.4±2.0	37.2±1.8	32.4±1.7	31.0±1.4	0.0±1.0	0.96
Macular GCL							
Central	45	12.0±1.0	15.5±1.9	13.8±1.9	18.5±1.7	2.3±0.7	0.002
Inner	45	34.1±1.8	42.9±1.7	40.1±1.3	36.7±2.5	0.7±1.0	0.52
Outer	45	25.4±0.7	27.0±0.8	26.7±0.5	23.9±0.9	0.6±0.4	0.15
Macular IPL							
Central	45	17.6±0.8	22.5±2.5	20.3±2.1	22.1±1.8	1.8±0.7	0.01
Inner	45	30.7±1.0	37.4±1.4	34.4±0.6	32.0±1.9	0.3±0.7	0.63
Outer	45	22.5±0.5	23.6±0.5	23.0±0.4	22.2±0.4	0.3±0.3	0.33
Macular GCC							
Central	43	40.7±2.2	48.4±4.3	43.6±4.4	54.1±4.1	4.8±1.7	0.005
Inner	43	86.1±3.1	103.5±3.3	97.1±2.3	91.8±5.1	1.7±2.0	0.38
Outer	43	77.4±2.8	87.8±2.7	82.1±2.4	77.2±2.7	0.7±1.4	0.63

*Adjusted for age and axial length.

CAREDS, Carotenoids in Age-Related Eye Disease Study; GCC, ganglion cell complex; GCL, ganglion cell layer; IPL, inner-plexiform layer; MPOD, macular pigment optical density; POAG, primary open-angle glaucoma; RNFL, retinal nerve fiber layer.

## Discussion

We assessed the relationship between MPOD and retinal thickness measured approximately 15 years later among a sample of older women with and without manifest POAG. We observed a significant positive association between baseline MPOD and macular GCC thickness in CAREDS2 within the central subfield, but not the inner or outer macular subfields. This finding was consistent among eyes with and without manifest POAG, despite considerable differences in GCC thickness between these groups. Additionally, eyes with manifest POAG showed a positive association between MPOD and superior and temporal peripapillary RNFL thickness.

Our results contribute to growing evidence of the association between MPOD and macular GCC thickness.^5 6 17^ Notably, we observed that MPOD at 0.5° was positively associated with GCC thickness in the central subfield, but not the inner and outer macular subfields. This may reflect higher MPOD in the fovea compared with the peripheral macula.^27^ Consequently, it is possible that MPOD may be a risk factor for foveal-involved POAG (causing paracentral vision loss), but not other types of glaucoma. This is consistent with the results from Siah *et al*^5^ that showed glaucomatous eyes with foveal GCC thinning (vs those without foveal GCC thinning) had nearly 50% lower MPOD in an Irish case–control study. In addition, Ji *et al*^6^ observed that MPOD was positively correlated with macular GCC thickness among eyes with POAG and those from age-matched controls in a small Chinese cross-sectional study. Most recently, Nagai *et al*^17^ reported that MPOD was positively correlated with macular GCC volume among healthy eyes from young Japanese participants (aged 22–48 years).

We also found an association between MPOD and thickness of the superior and temporal peripapillary RNFL quadrants in glaucomatous eyes, but not among healthy eyes. Prior small, cross-sectional studies have provided limited evidence regarding this relationship.^5 6^ Ji *et al* found no statistically significant associations between MPOD and peripapillary RNFL thickness^6^ except for a marginal association between MPOD and temporal RNFL thickness among glaucomatous eyes. Notably, this study did not include adjustment for age or axial length, known predictors of peripapillary RNFL thickness, which may explain the difference in our results. Siah *et al* reported a positive correlation between MPOD (0.5°) and peripapillary RNFL thickness in the inferior quadrant among patients with glaucoma, although this finding did not reach statistical significance.^5^ Glaucoma preferentially affects the superior and inferior quadrants of the peripapillary RNFL, which makes the association of these regions with MPOD notable.^4^ Peripapillary RNFL and GCC thinning are often detectable in early glaucoma before detectable visual field defects.^4^ Thus, our findings relating MPOD to peripapillary RNFL and GCC thickness support the hypothesis that low MPOD may contribute to or serve as a biomarker of glaucoma.

There are several plausible biological mechanisms that may underlie the association between MPOD and glaucomatous structural changes. Under the ‘protective’ hypothesis, macular pigment may mitigate the age-related decline in neural retinal thickness by reducing oxidative stress that contributes to neural cell death.^28^ L/Z promote antioxidant defenses through multiple pathways including direct scavenging of free radicals,^29 30^ suppressing proinflammatory signalling pathways,^31^ and filtering short-wavelength blue light.^32^ L/Z are also incorporated into cell membranes and increase their rigidity,^12^ and consequently may provide structural support for RGCs under elevated IOP. Alternatively, under the ‘structural’ hypothesis, greater retinal thickness in the fovea may facilitate accumulation of macular pigment by providing additional binding sites for L/Z. In a study of 11 individuals with Stargardt disease, Aleman *et al*^33^ reported that greater retinal thickness was associated with greater likelihood for increasing MPOD with L/Z supplementation.^33^ Likewise, thinning of the inner retina in glaucoma may contribute to lower MPOD (ie, reverse causation). Our results provide important clues concerning the underlying biological relationship between MPOD and manifest POAG, and support ongoing clinical trials to determine whether increasing MPOD through L/Z intake or low-cost supplementation may be effective in preventing POAG development or progression.^34–36^

Limitations of our study include that participants were older women and predominantly white and non-Hispanic. Thus, our results may not generalise to those who are younger, male or from other racial/ethnic groups. In addition, our statistical power may have been limited due to the relatively small sample size in the subgroup with manifest POAG. Yet, we did find significant associations, which supports the robustness of these relationships. Further, our results may have been affected by survival bias due to loss to follow-up and mortality, which was associated with slightly lower MPOD at CAREDS baseline that was not statistically significant (p=0.08).^37^ Since assessment of glaucoma measures (eg, RNFL OCT and visual field testing) were not performed at CAREDS baseline, we were unable to assess for progression of glaucomatous changes. We also cannot rule out the possibility that glaucomatous changes to the retina influenced MPOD measurements at CAREDS2 baseline. However, only 20.9% of participants with manifest POAG at CAREDS2 had at least one eye with a cup to disc ratio ≥0.6 at CAREDS baseline, an indicator of possible glaucomatous optic neuropathy.^38^ Finally, as with any observational study, our results may be affected by residual confounding. However, the similarity of our findings with those from prior smaller, case–control studies support the associations identified.

In conclusion, we observed a positive association between baseline MPOD and central subfield GCC thickness measured approximately 15 years later in eyes with and without manifest POAG. In addition, MPOD was positively associated with the thickness of the superior and temporal quadrants within the peripapillary RNFL among eyes with manifest POAG. Our results linking low MPOD to retinal layers that are structural indicators of early glaucoma provide further evidence that L/Z may provide protection against manifest POAG. Additional studies, including clinical trials, are needed to further elucidate the relationship between MPOD and the integrity of retinal layers associated with glaucoma, which may facilitate the development of novel interventions for manifest POAG.

## Data Availability

Data are available upon reasonable request. The data are available upon reasonable request to those who have obtained all required approvals from the Women’s Health Initiative and the University of Wisconsin Health Sciences Institutional Review Board.
