# Supplementary figures and images for: Association of macular pigment optical density with retinal layer thicknesses in eyes with and without manifest primary open-angle glaucoma
Source: BMJ Open Ophthalmol. 2023 Oct 27;8(1):e001331. doi: 10.1136/bmjophth-2023-001331 (PMC10619120; doi:10.1136/bmjophth-2023-001331)

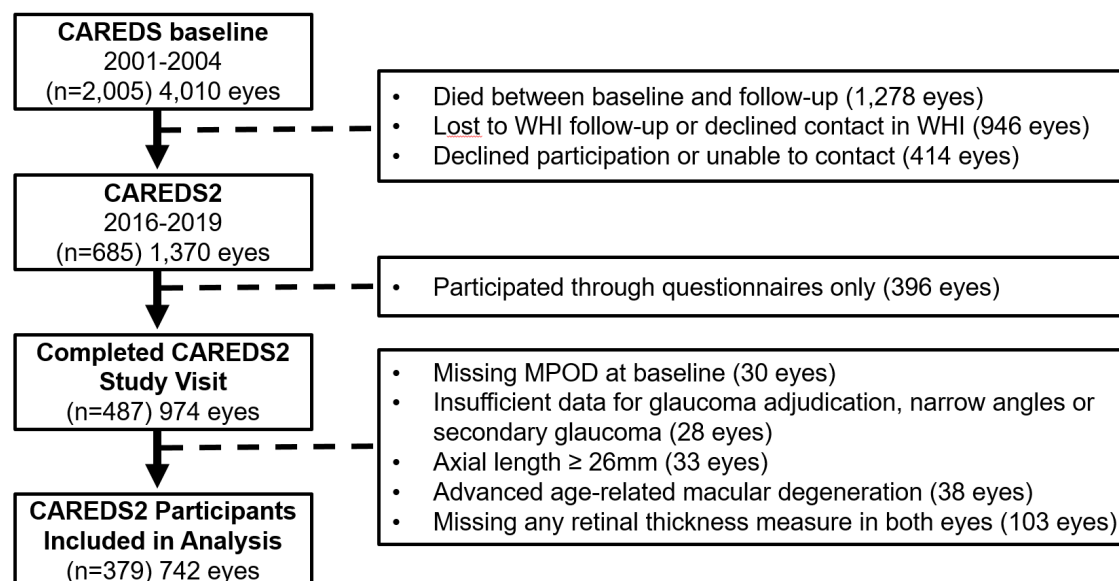

Supplement: Supplementary data [file bmjophth-2023-001331supp001.pdf]

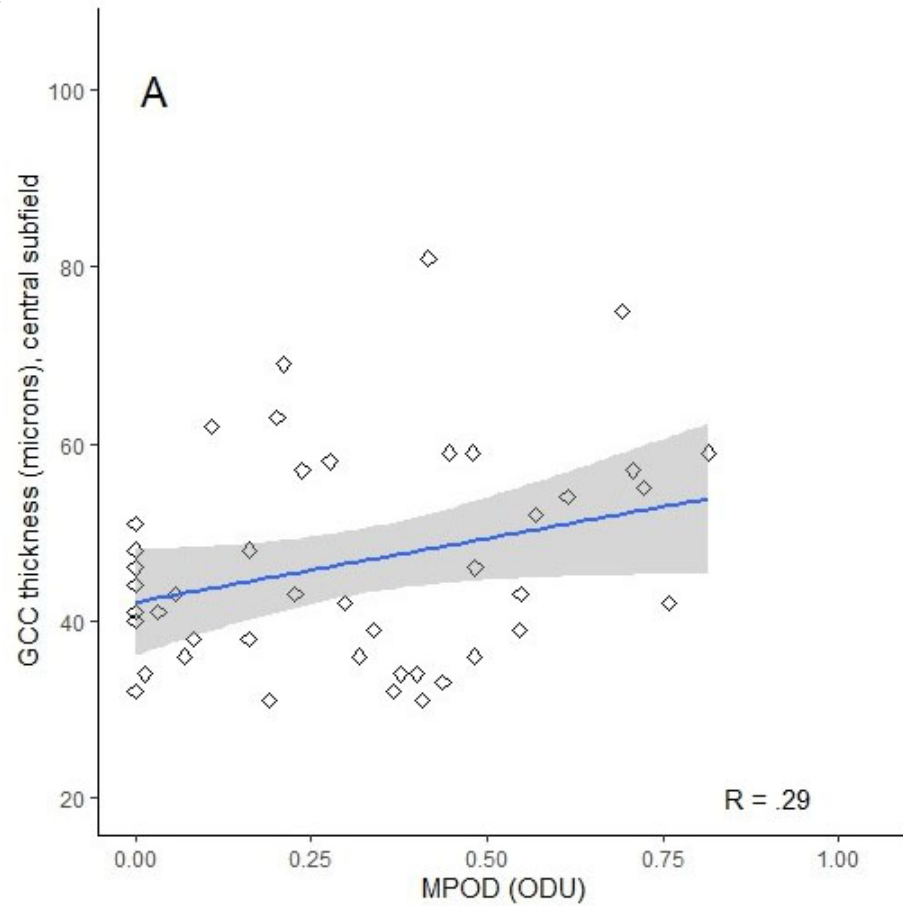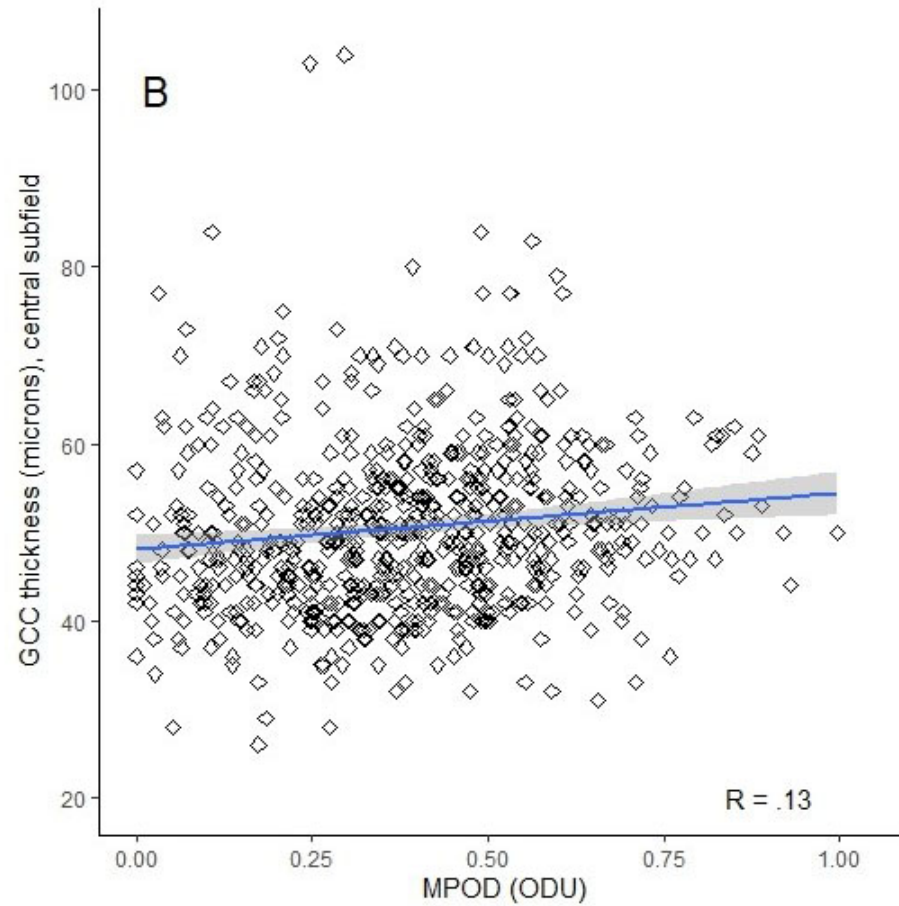

Supplement: Supplementary data [file bmjophth-2023-001331supp002.pdf]
