# Supplementary material for: Association of macular pigment optical density with retinal layer thicknesses in eyes with and without manifest primary open-angle glaucoma
Source: BMJ Open Ophthalmol. 2023 Oct 27;8(1):e001331. doi: 10.1136/bmjophth-2023-001331 (PMC10619120; doi:10.1136/bmjophth-2023-001331)
Supplement: Supplementary data [file bmjophth-2023-001331supp003.pdf]

Supplementary Table S1. Characteristics of CAREDS participants included and excluded in the analysis (N=2,005)

Characteristics*	All CAREDS participants (n = 2005)	Included (n = 379)	Excluded (n = 1,626)	*p*-value
Age (years) - CAREDS baseline	69.7±0.2	65.4±0.3	70.7±0.2	< .001
Race/Ethnicity				.84
Native American	0.0%	0.0%	0.1%	
Asian	0.7%	0.9%	0.7%	
Native Hawaiian/Pacific Islander	0.0%	0.0%	0.1%	
Black	1.0%	1.5%	1.1%	
White	97.1%	97.0%	96.7%	
More than one race	0.8%	0.4%	1.0%	
Unknown/not reported	0.3%	0.1%	0.4%	
Ethnicity				.93
Non-Hispanic	99.5%	98.6%	99.4%	
Hispanic	0.4%	1.4%	0.4%	
Unknown	0.1%	0.0%	0.1%	
Education				< .001
High school graduate or less	22.1%	13.9%	24.0%	
College graduate	47.8%	48.4%	47.8%	
Post-college education	30.0%	37.7%	28.1%	
Self-reported annual household income ≥ $75,000 - WHI baseline	17.2%	24.8%	15.3%	< .001
Pack years smoked - CAREDS baseline				.15
Never smoker	58.3%	62.4%	57.8%	
< 7 pack years	22.3%	24.0%	22.3%	
≥ 7 pack years	19.4%	13.6%	19.9%	
Intraocular lens – CAREDS baseline	16.0%	11.0%	16.6%	.04
Waist circumference (inches) - CAREDS baseline	34.71±0.12	33.9±0.3	34.9±0.1	.003
Body mass index (kg/m^2) - CAREDS baseline	28.0±0.1	27.1±0.3	28.2±0.1	.001
Self-reported hypertension – CAREDS baseline	28.2%	28.9%	29.0%	.18
Self-reported diabetes - CAREDS baseline	7.1%	4.2%	7.8%	.02
Self-reported glaucoma - CAREDS baseline	6.1%	4.2%	6.4%	.32
MPOD - CAREDS baseline (ODU) †	0.36±0.01	0.38±0.01	0.36±0.01	.08

*Values are as mean ± SE for continuous variables and percentages for categorical variables.
† Data presented from the right eye only
Abbreviations: CAREDS - Carotenoids in Age-Related Eye Disease Study; WHI - Women's Health Initiative; ODU – optical density units

Supplementary Table S2. Participant characteristics by manifest POAG status at CAREDS2 (N=379)

Characteristics*	No Manifest POAG (n = 347)	Manifest POAG (n = 32)	*p*-value
Age (years)	65.2±0.3	67.8±1.0	.01
Race			.53
Asian	0.6%	1.9%	
Black	0.6%	5.0%	
White	98.2%	93.1%	
More than one race	0.3%	0.0%	
Unknown/not reported	0.3%	0.0%	
Ethnicity			.99
Non-Hispanic	99.4%	100.0%	
Hispanic	0.6%	0.0%	
Education			.93
High school graduate or less	13.5%	5.8%	
College or vocational training	46.9%	57.8%	
Post college	39.6%	36.4%	
Household Income			.74
< $75,000	72.3%	72.2%	
> $75,000	27.7%	27.8%	
Pack years smoked			.03
Non-smoker	56.9%	74.1%	
< 7 pack years	24.2%	25.9%	
≥ 7 pack years	18.9%	0.0%	
Intraocular lens implantation*	5.7%	18.6%	.03
Intraocular pressure (mmHg)*	14.4±0.2	14.7±0.6	.58
Axial length (mm)*	23.6±0.1	23.8±0.2	.22
Corneal thickness (μm)*	558.5±1.9	554.5±6.5	.55
Waist circumference (in.)	34.1±0.3	33.6±0.9	.63
Body mass index (kg/m^2)	27.5±0.3	26.8±0.9	.44
Self-reported hypertension	21.2%	28.3%	.74
Self-reported diabetes	3.8%	1.9%	.76
Cup to disc ratio ≥ 0.6 – CAREDS baseline	2.5%	20.9%	< .001
MPOD – CAREDS baseline (ODU) †	0.39±0.01	0.31±0.04	.03

*Values for ocular characteristics are shown only for the right eye, except for cup to disc ratio ≥ 0.6 at CAREDS baseline, which refers to whether this characteristic was present in at least one eye

† Data presented from the right eye only

Abbreviations: CAREDS - Carotenoids in Age-Related Eye Disease Study; MPOD - macular pigment optical density; POAG – primary open-angle glaucoma; SE – standard error

Supplementary Table S3. Retinal layer thickness* by quartile of macular pigment optical density (MPOD) at CAREDS baseline among eyes without manifest POAG, excluding participants using lutein and zeaxanthin supplements (≥ 1 mg/day) prior to CAREDS2 (N = 302)

		**MPOD – CAREDS baseline, 0.5° (optical density units)**					
**Retinal layer thickness*** (μm, mean ± SE)	**Number of Eyes**	**Quartile 1 (0.00-0.23)**	**Quartile 2 (0.23-0.38)**	**Quartile 3 (0.38-0.51)**	**Quartile 4 (0.51-1.00)**	**β ± SE (1-SD increase)**	***P*-trend**
**Peripapillary RNFL**							
Average	535	94.2 ± 1.0	93.8 ± 1.1	93.1 ± 1.1	93.9 ± 1.0	-0.2 ± 0.5	.64
Inferior	535	121.2 ± 1.5	121.8 ± 1.7	120.7 ± 1.8	121.4 ± 1.6	-0.2 ± 0.8	.83
Superior	535	111.7 ± 1.6	111.2 ± 1.6	110.0 ± 1.6	113.1 ± 1.5	0.5 ± 0.8	.55
Nasal	535	72.6 ± 1.3	72.5 ± 1.3	70.7 ± 1.3	72.1 ± 1.2	-0.5 ± 0.7	.42
Temporal	535	71.3 ± 1.3	69.9 ± 1.5	71.0 ± 1.2	68.8 ± 1.3	-0.7 ± 0.7	.27
**Macular RNFL**							
Central	566	13.1 ± 0.3	12.7 ± 0.2	13.0 ± 0.2	13.7 ± 0.3	0.2 ± 0.1	.12
Inner	566	23.7 ± 0.3	23.1 ± 0.3	23.6 ± 0.3	23.5 ± 0.3	0.0 ± 0.1	.71
Outer	564	40.0 ± 0.6	38.5 ± 0.6	39.7 ± 0.6	39.3 ± 0.6	-0.1 ± 0.3	.68
**Macular GCL**							
Central	565	15.4 ± 0.5	15.0 ± 0.4	16.1 ± 0.4	17.1 ± 0.4	0.6 ± 0.2	.004
Inner	566	46.1 ± 0.5	45.7 ± 0.6	46.2 ± 0.5	46.7 ± 0.5	0.3 ± 0.3	.27
Outer	564	30.3 ± 0.3	30.2 ± 0.3	30.5 ± 0.4	30.3 ± 0.3	0.1 ± 0.2	.52
**Macular IPL**							
Central	567	20.7 ± 0.4	20.4 ± 0.3	21.4 ± 0.3	22.5 ± 0.4	0.7 ± 0.2	< .001
Inner	567	38.0 ± 0.3	37.9 ± 0.4	38.1 ± 0.3	38.6 ± 0.3	0.3 ± 0.1	.07
Outer	566	25.5 ± 0.2	25.6 ± 0.2	25.6 ± 0.2	25.8 ± 0.2	0.2 ± 0.1	.16
**Macular GCC**							
Central	555	48.5 ± 1.0	47.6 ± 0.9	50.3 ± 0.9	53.0 ± 1.1	1.7 ± 0.5	.001
Inner	556	107.5 ± 0.9	106.5 ± 1.1	107.8 ± 1.0	108.7 ± 0.9	0.6 ± 0.5	.23
Outer	555	95.4 ± 0.9	94.2 ± 1.0	95.8 ± 1.1	95.4 ± 0.9	0.2 ± 0.5	.65

*Adjusted for age and axial length

Abbreviations: CAREDS - Carotenoids in Age-Related Eye Disease Study; GCC - ganglion cell complex; GCL - ganglion cell layer; IPL - inner-plexiform layer; RNFL - retinal nerve fiber layer; SE - standard error; SD – standard deviation

Supplementary Table S4. Retinal layer thickness* by quartile of macular pigment density (MPOD) at CAREDS baseline among eyes with manifest POAG, excluding participants using lutein and zeaxanthin supplements (≥ 1 mg/day) prior to CAREDS2 (N = 25)

		**MPOD – CAREDS baseline, 0.5° (optical density units)**					
**Retinal layer thickness*** (µm, mean ± SE	**Number of Eyes**	**Quartile 1 (0.00-0.07)**	**Quartile 2 (0.08-0.30)**	**Quartile 3 (0.32-0.48)**	**Quartile 4 (0.48-0.81)**	**β ± SE (1-SD increase)**	***P*-trend**
**Peripapillary RNFL**							
Average	33	64.8 ± 3.9	74.0 ± 2.2	74.2 ± 3.5	75.9 ± 2.0	3.3 ± 1.5	.03
Inferior	33	74.7 ± 6.5	90.7 ± 5.2	83.9 ± 5.8	75.3 ± 4.9	-0.1 ± 2.8	.98
Superior	33	72.1 ± 4.0	81.5 ± 2.8	94.3 ± 5.3	93.5 ± 3.8	7.0 ± 2.4	.004
Nasal	33	56.8 ± 3.3	58.2 ± 3.4	57.0 ± 6.7	61.8 ± 2.6	1.4 ± 1.6	.40
Temporal	33	56.0 ± 2.7	65.8 ± 2.4	61.1 ± 2.4	73.2 ± 3.5	5.0 ± 1.5	.001
**Macular RNFL**							
Central	37	11.4 ± 0.8	13.4 ± 0.9	9.8 ± 1.1	13.1 ± 0.8	0.4 ± 0.5	.38
Inner	37	21.1 ± 0.7	24.5 ± 0.9	22.4 ± 0.7	22.8 ± 0.6	0.5 ± 0.4	.20
Outer	37	29.6 ± 2.2	38.0 ± 1.8	31.7 ± 2.2	32.2 ± 1.6	0.2 ± 1.1	.84
**Macular GCL**							
Central	37	12.8 ± 0.9	16.9 ± 1.7	12.2 ± 1.8	17.2 ± 0.7	1.2 ± 0.6	.03
Inner	37	35.2 ± 2.0	43.9 ± 1.8	40.1 ± 1.9	39.0 ± 2.7	0.9 ± 1.2	.44
Outer	37	25.6 ± 0.8	27.3 ± 0.9	26.8 ± 0.7	25.2 ± 0.8	-0.2 ± 0.4	.56
**Macular IPL**							
Central	37	18.2 ± 0.7	23.9 ± 2.5	17.4 ± 1.3	21.5 ± 1.5	1.0 ± 0.6	.09
Inner	37	31.3 ± 1.1	38.2 ± 1.5	33.8 ± 1.0	33.6 ± 1.7	0.4 ± 0.7	.53
Outer	37	22.4 ± 0.5	23.9 ± 0.6	22.9 ± 0.5	22.4 ± 0.6	-0.2 ± 0.3	.45
**Macular GCC**							
Central	36	42.2 ± 2.1	51.7 ± 3.7	39.6 ± 4.3	52.3 ± 2.5	2.7 ± 1.5	.07
Inner	36	87.6 ± 3.4	105.4 ± 3.3	96.3 ± 3.4	96.6 ± 4.6	2.2 ± 2.1	.30
Outer	36	77.6 ± 3.0	89.2 ± 2.8	81.3 ± 3.1	80.0 ± 2.6	-0.2 ± 1.6	.90

*Adjusted for age and axial length

Abbreviations: CAREDS - Carotenoids in Age-Related Eye Disease Study; GCC - ganglion cell complex; GCL - ganglion cell layer; IPL - inner-plexiform layer; RNFL - retinal nerve fiber layer; SE - standard error; SD – standard deviation
